# Targeting the p53 Pathway in CLL: State of the Art and Future Perspectives

**DOI:** 10.3390/cancers13184681

**Published:** 2021-09-18

**Authors:** Marwan Kwok, Angelo Agathanggelou, Nicholas Davies, Tatjana Stankovic

**Affiliations:** 1 Institute of Cancer and Genomic Sciences, University of Birmingham, Birmingham B15 2SY, UK; a.agathanggelou@bham.ac.uk (A.A.); n.j.davies@bham.ac.uk (N.D.); 2 Centre for Clinical Haematology, Queen Elizabeth Hospital Birmingham, Birmingham B15 2SY, UK

**Keywords:** p53, ATM, MDM2, CDK, ATR, PARP, DNA-PK, USP7, synthetic lethality, immunity

## Abstract

**Simple Summary:**

The p53 pathway plays a fundamental role in preventing the accumulation of unrepaired DNA damage. Unsurprisingly, cancer cells often break this protective barrier by inactivating either p53 itself or other proteins along this pathway, such as ATM. This facilitates the acquisition of further alterations that are required for unperturbed cancer growth and the development of chemoresistance. Over the years, there has been substantial scientific investment in creating new cancer therapies that can overcome functional loss of the p53 pathway. In this review, we will discuss different therapeutic approaches towards achieving this goal within the context of chronic lymphocytic leukemia (CLL), a cancer type with a p53-deficient subset that remains challenging to treat.

**Abstract:**

The p53 pathway is a desirable therapeutic target, owing to its critical role in the maintenance of genome integrity. This is exemplified in chronic lymphocytic leukemia (CLL), one of the most common adult hematologic malignancies, in which functional loss of p53 arising from genomic aberrations are frequently associated with clonal evolution, disease progression, and therapeutic resistance, even in the contemporary era of CLL targeted therapy and immunotherapy. Targeting the ‘undruggable’ p53 pathway therefore arguably represents the holy grail of cancer research. In recent years, several strategies have been proposed to exploit p53 pathway defects for cancer treatment. Such strategies include upregulating wild-type p53, restoring tumor suppressive function in mutant p53, inducing synthetic lethality by targeting collateral genome maintenance pathways, and harnessing the immunogenicity of p53 pathway aberrations. In this review, we will examine the biological and clinical implications of p53 pathway defects, as well as our progress towards development of therapeutic approaches targeting the p53 pathway, specifically within the context of CLL. We will appraise the opportunities and pitfalls associated with these therapeutic strategies, and evaluate their place amongst the array of new biological therapies for CLL.

## 1. Introduction

Chronic lymphocytic leukemia (CLL) is a malignant clonal proliferative disorder of mature CD5^+^ B lymphocytes that accumulate within the peripheral blood, lymphoid tissues, and bone marrow. A characteristic feature of CLL is its clinical heterogeneity, with highly divergent clinical courses ranging from indolent or even spontaneously regressing trajectories [1,2], to rapidly progressive trajectories, in which CLL remains incurable and life-limiting despite recent advances in targeted therapy and immunotherapy [3]. Clinical heterogeneity in CLL reflects its biological heterogeneity with a lack of ubiquitous disease-defining genomic lesions that govern malignant transformation [4]. Instead, the development and progression of CLL is contingent upon successive acquisition of malignant properties including apoptotic resistance, proliferative signaling, mutational burden, and genomic instability [3]. These properties, acquired through a range of lesions underpinned by genetic clonal evolution within a permissive tumor microenvironment, confer progressive survival and proliferative advantage [5].

Of the genetic aberrations that are observed in CLL, those affecting the p53 pathway are among the most important in their contribution to CLL progression. p53 is a transcription factor encoded by the tumor suppressor gene *TP53*, the canonical function of which relates to its fundamental role in the maintenance of genome integrity [6]. p53 transcriptionally regulates the expression of genes involved in cell cycle control, DNA repair, and apoptosis in response to cellular DNA damage, with salvageable lesions resulting in cell cycle arrest and DNA repair, and irreparable lesions resulting in apoptosis [7]. In this respect, the phosphatidyl inositol 3′ kinase-related kinases (PIKK), ataxia telangiectasia mutated (ATM), and ataxia telangiectasia and Rad3-related (ATR), acting upstream of p53, are master regulators of response to incipient DNA insults. ATM mediates the repair of DNA double-strand breaks through homologous recombination [8,9], whereas ATR mediates the resolution of predominantly single-strand DNA lesions that impede DNA replication and induce replication stress [10]. Both kinases converge onto p53, stabilizing and activating it through direct p53 phosphorylation, and indirectly through the repression of the ubiquitin ligase mouse double minute 2 (MDM2) that negatively regulates p53 [10,11,12]. Through concerted effects of ATM, ATR, and p53, cells are protected from the accumulation of deleterious DNA alterations that underscore genomic instability, malignant transformation, subclonal diversification, and clonal evolution. 

In CLL, disruptions to the p53 pathway arise primarily through deletions of chromosomes 17p and 11q containing *TP53* and *ATM,* respectively, and from deleterious mutations affecting these genes [13,14,15,16]. Monoallelic 17p or 11q deletions are amongst the most frequent cytogenetic aberrations in CLL, occurring in >15% and >5%, respectively, of CLL patients [17]. Monoallelic del(17p) or del(11q) may co-occur with mutation in the remaining *TP53* or *ATM* allele, resulting in biallelic *TP53* or *ATM* loss that abolishes the protective barrier against DNA damage and genomic instability [14,15,18,19]. Moreover, dominant negative effects exerted by monoallelic *TP53* or *ATM* mutations on the residual wild-type *TP53* or *ATM* allele could also abrogate protective ATM/p53 signaling even in the absence of biallelic *TP53* or *ATM* defects [14,15]. Finally, recent discoveries of transcription-independent [20] and noncanonical [21] p53 functions, as well as oncogenic gain-of-function properties [22,23] of specific *TP53* mutations, have broadened our understanding of the role of the p53 pathway beyond genome maintenance to include such diverse processes as metabolism and autophagy, as well as antioxidant, immune, and inflammatory response [21]. It is unsurprising, therefore, that *TP53* and *ATM* alterations are enriched in clinically advanced and treatment refractory CLL consequent upon natural selection of the ‘fittest’ CLL subclones harboring such defects (Table 1) [15,19,24].

The adverse clinical impact of p53 pathway defects justifies the development of novel therapeutic approaches targeting this pathway. This endeavor, however, is fraught with difficulty due to the indispensable nature of p53 both for normal cells and for countering malignant progression. In recent years, several therapeutic avenues targeting the p53 pathway have been developed, leveraging our understanding of this pathway and the cellular dependencies resulting from p53 pathway defects. In this review, we will appraise recent advances in the utilization of p53 pathway defects, both as prognostic biomarkers and as novel therapeutic targets for CLL.

## 2. p53 Pathway Defects as Adverse Prognostic Biomarkers in CLL

The advents of next-generation sequencing and longitudinal analysis of CLL clonal dynamics have provided valuable insight into how p53 pathway defects emerge and evolve alongside other genomic alterations (Table 1) [25,26,30,31,32]. These studies have demonstrated that *TP53* and *ATM* defects can be either clonal or subclonal, with the former representing genomic events that have arisen early during CLL development, while the latter corresponds to later events that emerge as a result of clonal selection pressures [25]. Indeed, deletions and mutations of *TP53* and *ATM* have been reported in monoclonal B-cell lymphocytosis (MBL) and early-stage CLL, albeit at much lower frequencies than in patients with more clinically advanced disease [33,34]. Nevertheless, even at such an early stage, the presence of these aberrations confers adverse prognostic impact and portends disease progression [34], reflecting the biological consequences of p53 or ATM loss. Among *TP53* mutations, the most predominant type is missense, followed by frameshift mutations, in-frame deletions, and mutations affecting the exon/intron splicing site. The mutations tend to cluster within the p53 DNA binding domain and are evenly distributed between exons 5 to 8. Monoallelic *TP53* alterations exert a less detrimental prognostic impact, compared to biallelic alterations. Among monoallelic alterations, single missense mutation rather than single deletion undergo the process of clonal selection in CLL cells [15]. In comparison, *ATM* mutations are distributed across the whole gene, comprising a mixture of different alteration subtypes [13,14,19]. Furthermore, monoallelic *TP53* or *ATM* loss provides selective pressure for the subsequent loss of the remaining *TP53* or *ATM* allele [15,18], resulting in marked CLL growth acceleration secondary to an abrogation of cell cycle and apoptotic control [31]. Later genomic events may alternatively reflect subclonal outgrowth resulting from therapeutic bottlenecks during CLL treatment [26], such as the selection and expansion of small *TP53*-mutated subclones that carry similar prognostic impact to clonal *TP53* aberrations [24,28].

Given the loss of apoptotic response to DNA damage, CLL with *TP53* or *ATM* deletions or mutations predictably confers inferior survival outcomes with conventional chemotherapy [16,18,19,27]. The addition of anti-CD20 monoclonal antibodies such as rituximab or obinutuzumab to chemotherapeutic regimens appears to overcome adverse prognosis associated with monoallelic del(11q) or *ATM* mutation [26,29], although its impact on CLL with biallelic *ATM* inactivation remains to be ascertained. Clinical benefit with chemoimmunotherapy may be predicated upon the eradication of minimal residual disease (MRD), which we and others have shown to be important for determining response durability, particularly in the frontline setting [35,36]. Conceivably, patients with more profound remissions after treatment have a smaller pool of residual CLL cells from which chemoresistant subclones harboring p53 pathway defects could arise or become enriched, and are therefore less likely to relapse.

The past decade has witnessed the development of B-cell receptor (BCR) signaling inhibitors (e.g., ibrutinib) and Bcl-2 inhibitors (e.g., venetoclax) that target essential cellular dependencies of CLL. Notwithstanding remarkable improvement in clinical outcome in comparison with chemotherapy-based treatments, *TP53* defects remain predictors for shortened response durability. For instance, 5-year follow-up analysis of the pivotal PCYC-1102 trial of ibrutinib showed a median progression-free survival (PFS) in the relapsed/refractory setting of 26 months for del(17p) patients compared to 51 months for all patients, with del(17p) remaining a significant predictor of PFS in multivariate analysis [37]. Although the larger RESONATE trial showed only a trend towards inferior outcome for del(17p) or *TP53* mutation [38], patients harboring biallelic *TP53* defects displayed significantly worse PFS [39]. With respect to the venetoclax plus rituximab combination, the MURANO trial at 4-year follow-up demonstrated significantly lower rates of undetectable MRD and inferior PFS in patients with del(17p) [40]. Likewise, del(17p) and *TP53* mutation both predicted for inferior PFS within the CLL14 trial, which evaluated the venetoclax plus obinutuzumab combination [41]. *TP53* defect, with its associated predisposition to genomic instability and clonal complexity [42,43,44], likely facilities the development of resistance mechanisms such as *BTK* or *PLCγ2* mutations in the case of ibrutinib [45,46]; and *BCL-2* mutation, Mcl-1 upregulation, or metabolic reprogramming in the case of venetoclax [47,48]. Gene-expression profiling has also suggested a reduced dependence on BCR signaling in *TP53*-mutated CLL [49]. Moreover, patients under ibrutinib or venetoclax therapy continue to be at risk of Richter transformation, with *TP53* disruption and DNA damage response (DDR) alterations featuring prominently in this process [50,51,52].

Accordingly, new treatments for CLL, particularly those targeting p53 pathway defects that constitute a major source of therapeutic resistance, are still unquestionably needed. The remainder of this article will highlight several past, present, and emerging strategies for p53 pathway targeting in CLL. These include the upregulation of wild-type p53, the restoration of tumor-suppressive function in mutant p53, the induction of synthetic lethality through targeting collateral genome maintenance pathways, and strategies harnessing the immunogenicity of p53-pathway aberrations (Table 2).

## 3. Inhibiting the Inhibitor: Targeting MDM2 to Upregulate Wild-Type p53

One of the earliest p53-based therapeutic strategies emanates from our recognition that p53 activation is a tightly regulated physiological process. This is achieved primarily through p53′s interaction with MDM2, the major cellular p53 antagonist that suppresses p53 activity through mediating its ubiquitin-dependent proteasomal degradation [75]. MDM2 is overexpressed in >20% of CLL patients through a single nucleotide polymorphism (SNP; T > G) at position 309 in the promoter region of *MDM2* [76,77], which serves as an additional mechanism through which CLL cells can attenuate p53 regulation. MDM2 overexpression resulting from this SNP confers inferior prognosis in CLL [76,77], underscoring the importance of the MDM2–p53 interaction for CLL biology. The importance of this interaction is also evidenced by *RPS15* mutations, seen in some patients with CLL. *RPS15* is a gene that encodes the ribosomal protein S15, a component of the 40S ribosomal subunit. It stabilizes p53 through interfering with the MDM2–p53 interaction. *RSP15* mutation results in loss of RPS15 function and impaired p53 stability, leading to inferior survival outcome in CLL patients with mutated *RPS15* compared to their counterpart with wild-type *RPS15* [78].

**Figure 1:**
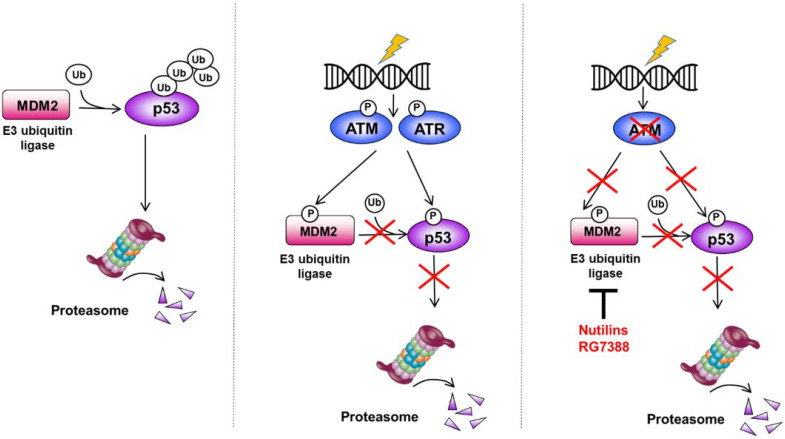
Targeting MDM2 to upregulate wild-type p53. Under baseline conditions, the E3 ubiquitin ligase MDM2 targets p53 for proteasome degradation by ubiquitination (**left panel**). Following induction of DNA double- or single-strand breaks, ATM and ATR kinase are respectively activated, leading to phosphorylation of both MDM2 and p53, with consequent inhibition of MDM2 activity and p53 upregulation (**middle panel**). In the absence of ATM function, MDM2 continues to induce p53 degradation, despite the presence of DNA damage. This effect can be counteracted by MDM2 inhibitors such as nutlins or RG7388 (**right panel**).

The MDM2–p53 interaction therefore represents an attractive therapeutic target for CLL with functional p53, the inhibition of which would be expected to upregulate p53 levels, thereby enhancing tumor control (Figure 1) [79]. MDM2 inhibitors such as nutlins and RG7388 bind MDM2 in the p53-binding pocket, disrupting MDM2–p53 binding and hence stabilizing p53 [80]. Given the need for functional p53 to instigate downstream p53-dependent activity, one could reasonably speculate that such a strategy would be effective in cases in which p53 activity is repressed due to *ATM* disruption, MDM2 overexpression, or possibly monoallelic *TP53* deletion with residual wild-type p53 function. Indeed, MDM2 inhibition in primary CLL cells harboring *ATM* loss or MDM2 overexpression resulted in the effective induction of p53 target genes and p53-mediated apoptosis [53,54,55,56]. On the contrary, CLL samples with *TP53* defects exhibited variable sensitivity to MDM2 inhibitors depending on the nature of the *TP53* aberration and the proportion of CLL cells harboring it, with loss of p53 function correlating strongly with diminished sensitivity to MDM2 inhibition [53,55,56]. In addition to *TP53* status, DNA damage signaling has also been shown to influence sensitivity to MDM2 inhibitors, and tumors with both wild-type p53 as well as activated DDR signaling appear to benefit most from MDM2 inhibition [81].

The clinical activity of the MDM2 inhibitor RG7112 was assessed in a phase I trial in which RG7112 was administered to 20 patients with heavily pretreated CLL, of whom 6 had *TP53* defects [82]. Only modest clinical activity was observed, with one patient achieving partial response, whereas the majority had stable disease. However, the RG7112 trial evaluated drug doses that were substantially below the therapeutic range anticipated from preclinical studies, thus hampering the assessment of the inhibitor’s true clinical efficacy. As expected, patients with mutant *TP53* generally failed to achieve a clinical response [82].

There are two further caveats concerning the clinical use of MDM2 inhibitors. The first of these relates to their potential toxicity to normal cells from spontaneous p53 activity in the absence of negative regulation by MDM2 [83]. Reassuringly, the pattern of p53-dependent gene expression induced by MDM2 inhibition was found to be substantially different in CLL cells compared to normal blood cells, with predominantly cell-cycle arrest rather than apoptosis being elicited in normal cells at therapeutic MDM2 inhibitor concentrations [56]. Nevertheless, myelosuppression appears to be inevitable with clinical use of MDM2 inhibitors, and infections are the most frequently reported severe adverse events [82]. The other issue relates to the potential for selection of drug-resistant p53 mutants during MDM2 inhibitor use. Although this scenario has only been modelled within preclinical studies [84,85,86], such a possibility should be considered as a potential mechanism underlying treatment refractoriness.

## 4. Confronting Mutant p53: Strategies to Restore the Tumor-Suppressive Function

Inactivating *TP53* mutation poses an impediment to the use of therapeutic strategies aimed at p53 upregulation. In view of this, there has been effort to devise strategies in order to restore wild-type p53 function. Approaches that have been studied in CLL include those that bypass nonfunctional p53 by acting downstream of p53 to regulate genome maintenance activities (e.g., cyclin-dependent kinase (CDK) inhibitors), and others that seek to structurally alter mutant p53 or otherwise attempt to deplete it (e.g., PRIMA-1 and HSP90 inhibitors, respectively). Each of these approaches will be discussed in turn (Table 2 and Table 3).

### 4.1. Inhibiting CDK to Restore Transcriptional Control of Apoptosis and Prosurvival Responses

**Figure 2:**
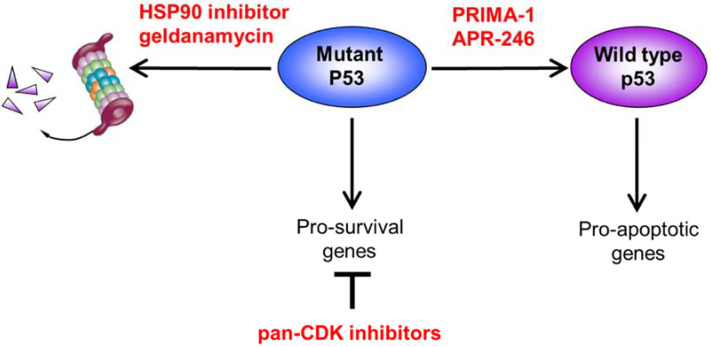
Strategies to restore the tumor-suppressive function of mutant p53. Wild-type p53 regulates the transcription of proapoptotic genes. In response to DNA damage, mutant p53 fails to activate proapoptotic genes. p53-independent ATM-dependent transcription leads to the activation of strong prosurvival signals. This effect can be counteracted by CDK inhibitors that suppress RNA polymerase II function. Other strategies to restore normal p53 function involve altering the configuration of mutant p53 by PRIMA-1 and APR-246, as well as its proteasomal degradation facilitated by HSP90 inhibition.

Acting as a transcription factor, p53 regulates a large network of genes that participate in genome maintenance. p53, for instance, transactivates *CDKN1A,* which in turn mediates p53-dependent G1/S cell-cycle arrest by binding to and inhibiting other CDKs [21]. Through phosphorylating substrates including p53, ATM also exerts a significant influence on genome regulation in response to DNA damage [9,12]. Our earlier work established distinct transcriptional profiles in *TP53*-mutant CLL as compared to *ATM* mutants [98]. While *TP53* and *ATM* mutants both showed compromised proapoptotic response to DNA insults, *TP53* mutants additionally exhibited an elevated survival response consequent upon p53-independent, ATM-mediated prosurvival activity [98]. On this basis, we subsequently demonstrated the potential clinical utility of the pan-CDK inhibitor roscovitine, which inhibits RNA polymerase II activity and downregulates genes involved in transcription and translation initiation, as well as DNA repair and cellular survival, including antiapoptotic genes such as *MCL1*, thus targeting the antiapoptotic and other prosurvival properties of p53-defective CLL [57]. Similar findings were observed with other pan-CDK inhibitors including flavopiridol, SNS-032, and dinaciclib, demonstrating effects of CDK inhibition beyond interruption of cell-cycle progression (Figure 2) [58,59,60].

Within preclinical studies, treatment with pan-CDK inhibitors resulted in profound cytotoxicity towards CLL cells, including *TP53* mutants [57,59,60,99,100]. Despite demonstrable differences in transcriptional regulatory effects on CLL versus normal cells [57], the first-generation CDK inhibitors roscovitine and flavopiridol displayed a narrow therapeutic window and low selectivity, thus potentially limiting their clinical use. Nevertheless, in a clinical study involving 42 CLL patients, flavopiridol successfully induced partial remissions in 45% of individuals, with tumor lysis syndrome being the main dose-limiting toxicity [87]. The second-generation inhibitors SNS-032 and dinaciclib were designed to improve therapeutic index, thereby reducing toxicity. Initial results of SNS-032 disappointingly showed only a single responder out of 19 CLL patients [88]. On the other hand, dinaciclib exhibited greater clinical activity, inducing partial responses in 28 of 52 patients with relapsed CLL in one study [89], and 8 of 20 patients in another [90], including in the del(17p) CLL subgroup, which showed similar response rates to the wider cohort. Tumor lysis syndrome and infections continued to be observed with dinacicib [89,90], albeit at lower frequencies and severity compared to flavopiridol, indicating that further work is needed to optimize this therapeutic approach for clinical use.

### 4.2. Altering Mutant p53 Conformation to Restore Wild-Type Function

*TP53* mutations often lead to aberrant protein folding, with resultant loss of DNA binding and transcription factor function. An appealing therapeutic strategy for *TP53*-mutant tumors is to restore the wild-type conformation and transcriptional activity of p53. In this regard, PRIMA-1 and its analog APR-246 (PRIMA-1^Met^) are compounds that bind to cysteine residues in p53 and promote the refolding of mutant p53, thereby restoring wild-type function and inducing p53 gene targets (Figure 2) [61,101]. These compounds also bind and deplete cellular glutathione that could trigger lipid peroxidative cell death in p53-defective tumors [102]. In CLL, PRIMA-1 and APR-246 have been shown to induce cytotoxicity regardless of *TP53* mutational status [61,62]. In two phase I studies of APR-246 in hematologic malignancies that included four treatment-refractory CLL patients, clinical response was observed in 1 of 3 patients assessed. APR-246 was well tolerated, with the most common adverse effects being neurological [91,103]. Clinical studies are ongoing to establish its clinical activity in combination with ibrutinib or venetoclax in *TP53*-mutant relapsed/refractory CLL (NCT04419389).

### 4.3. Destabilizing Mutant p53 through HSP90 Inhibition

The increasingly recognized gain-of-function properties of many mutant p53 proteins have provided rationale for alternative approaches that aim to deplete mutant p53 within tumor cells (Figure 2). The proper folding and stability of conformationally aberrant mutant p53 is dependent on heat shock protein 90 (HSP90), a molecular chaperone that binds mutant p53, among other client proteins [104]. HSP90 inhibition destabilizes mutant p53 and induces its degradation [63], but has also been reported to upregulate wild-type p53, although the mechanism underlying the latter is unclear [64]. The HSP90 inhibitor geldanamycin was shown to be cytotoxic to primary CLL cells, including those with *TP53* mutation [64]. Nevertheless, clinical data has been lacking for CLL, with clinical studies in other cancer types showing excessive toxicity. On the other hand, HSP90 inhibitors with improved pharmacological properties could generate renewed interest in this strategy [105]. In addition, the recent discovery that HSP90-dependent mutant p53 accumulation is sustained through the mevalonate-RhoA axis could provide impetus for the development of an alternative therapeutic strategy to destabilize mutant p53 involving suppression of this pathway [106]. 

## 5. Novel Synthetically Lethal Strategies Exploiting p53 Pathway Defects

Tumor cells possess intrinsically high levels of DNA damage, as well as oxidative and replicative stress [107,108]. This promotes genomic instability that facilitates tumor evolution and progression, but exacerbation of cellular stress can also be fatal to tumor cells. A novel concept for targeting p53 pathway defects, known as synthetic lethality [109,110], capitalizes on the pivotal role of the p53 pathway in stress response and DNA repair. Cells have evolved functional redundancies in which simultaneous routes exist to resolve cellular stress and DNA damage. Disruption to the p53 pathway renders tumor cells reliant on collateral pathways to ameliorate cellular stress and maintain genome integrity. It follows that where two independent pathways regulate an essential genome regulatory process, the absence of one pathway is compatible with cell survival, whereas the absence of both commits cells to death [109,110]. This functional addiction of p53- or ATM-defective tumor cells has therefore created vulnerabilities that allow for rational therapeutic targeting involving abolition of collaborating pathways to induce tumor cytotoxicity. Several of these therapeutic avenues have come to the fore in recent years, each of which will be discussed below.

### 5.1. Exacerbating Oxidative-Stress-Induced Cytotoxicity 

CLL cells are reliant on the p53 pathway and its fine-tuning role for resolution of oxidative stress [111]. p53 upregulates several important cellular antioxidants, including glutathione peroxidase and aldehyde dehydrogenase 4 (ALDH4) [112,113,114], and induces metabolic targets such as sestrins, glutaminase 2 (GLS2), and TIGAR that decrease intracellular reactive oxygen species (ROS) [115,116,117]. Conversely, in response to catastrophic levels of oxidative stress, p53 sensitizes tumor cells to lipid peroxidative cell death through the repression of *SLC7A11*, a major constituent of the cysteine/glutamate antiporter, resulting in inhibition of cysteine uptake and suppression of cellular glutathione production [118]. Mutant p53 also represses *SLC7A11* through binding to and entrapping the master antioxidant transcription factor NRF2 [102]. Tumor cells with *TP53* defects are therefore expected to exhibit enhanced susceptibility to oxidative stress overload. Indeed, preclinical studies demonstrate enhanced sensitivity of p53-defective CLL cells to phenethyl isothiocyanate (PEITC), which exacerbates oxidative stress to intolerable levels through induced depletion of cellular glutathione [65,66]. Moreover, treatment of transgenic CLL mice harboring the *TCL1*-Tg:*p53*^−/−^ genotype with PEITC prolonged survival of these animals [65].

CLL cells with *ATM* defects are likewise susceptible to lipid peroxidative cell death secondary to oxidative stress overload. Studies have suggested that ATM acts as a sensor for intracellular ROS, the oxidation of which induces ATM dimerization and activation of downstream cellular response, which likely includes the modulation of intracellular ROS levels, ROS-dependent autophagy, as well as DDR [119,120,121]. In particular, ATM and its substrate BRCA1 can mediate antioxidant activity through regulation of the pentose–phosphate pathway and NRF2, respectively [122,123]. ATM-null CLL cells display impaired binding of NRF2 to antioxidant response elements and defective NRF2-regulated gene expression, which result in reduced levels of cellular glutathione and elevated cellular ROS [67]. Consequently, CLL cells and murine models with an ATM-null phenotype are hypersensitive to pro-oxidants such as parthenolide, as evidenced in our previous work [67]. The clinical utility and benefit of such an approach in CLL, however, remains to be determined.

### 5.2. Targeting Replication Stress through ATR/Chk1 Inhibition

Replication stress arises when replication fork progression stalls due to replication obstacles created by unrepaired DNA damage or replication errors [108]. Under such circumstances, cells ordinarily respond by activating ATR. This activation permits temporary cessation of DNA replication via G2/M cell-cycle arrest effected through its downstream mediator Chk1 [124,125], as well as the removal of replication obstacles by repairing DNA lesions at sites of replication fork stall [10,126,127]. ATR also holds a paramount role in preventing the unrestrained initiation of DNA replication [128,129]. Functional inhibition of ATR exacerbates replication stress by permitting excessive unscheduled replication initiation that depletes cellular pools of nucleotides and replication proteins, and stalls replication as a result [130,131]. Continued unwinding of the DNA helix at sites of stalled replication results in the formation of long stretches of unreplicated single-stranded DNA that are susceptible to breakage and collapse, generating double-strand breaks (DSBs) that impose a requirement for ATM-mediated homologous recombination repair and p53-mediated G1/S arrest to prevent further replication perturbation [68,132].

In CLL, we showed that *ATM* and *TP53* defects are synthetically lethal with ATR loss (Figure 1) by constraining cells to repair large volumes of DSBs through nonhomologous end joining (NHEJ) [68], an error-prone, low-fidelity backup path that often results in further genomic aberrations [133]. Likewise, the loss of both G1/S and G2/M checkpoints due to the combined consequence of ATM/p53 and ATR loss permits unrestricted entry into mitosis despite damaged DNA, leading to mitotic cell death from the accumulation of such damage to catastrophic levels [134,135]. Our preclinical studies confirmed that treatment with the ATR inhibitor ceralasertib (AZD6738) induces selective cytotoxicity and chemosensitization of *TP53-* or *ATM*-defective CLL cells, as well as in CLL patient-derived xenograft models with biallelic *TP53* or *ATM* loss, in which treatment with ceralasertib led to a reduction not only in tumor load, but also in the proportion of CLL cells with these genetic defects [68]. In two separate studies, Chk1 inhibitors also displayed significant single-agent activity and capacity to induce chemosensitization of *TP53*-mutant CLL cells [69,70].

An early-phase trial of ceralasertib in CLL is ongoing (NCT03328273). However, one feature that might limit its potential utility as a single agent is its functional dependence upon cell proliferation, with quiescent CLL cells being relatively resistant to ATR inhibitors due to the downregulation of ATR in noncycling cells [68,136]. Therefore, although our in vitro data supported an additive to synergistic interaction between ceralasertib and ibrutinib [68], this should be interpreted with due consideration of the impact of ibrutinib-mediated suppression of CLL proliferation on ceralasertib sensitivity. Hence, it is important in clinical practice to ‘front-load’ with ceralasertib in order to adequately target the proliferative *TP53*-mutant CLL population prior to introducing BCR signaling inhibitors. In this regard, as a monotherapy, Chk1 inhibitors might hold greater promise, given their demonstrable in vitro efficacy in quiescent as well as proliferating CLL cells [69], although in the proliferating population, ATR inhibitors acting upstream of Chk1 might produce more wide-ranging antitumor effects [10,130,131].

Published trials involving ceralasertib in solid tumors showed a generally encouraging toxicity profile, with myelosuppression being the major dose-limiting toxicity when used with chemotherapy [137,138]. However, one must be mindful of the potential longer-term effects of sublethal targeting of tumor cells with ATR/Chk1 inhibitors that may promote genomic instability and clonal evolution, if these cells are allowed to accumulate replication stress-induced DNA damage but escape death [139]. Patient selection is therefore of utmost importance to limit the use of these inhibitors to individuals who are predicted to be hypersensitive to ATR/Chk1 inhibition by virtue of their CLL genetic vulnerability that involves different genetic causes of replication stress.

### 5.3. Targeting PARP, DNA-PK, and USP7 Addiction 

**Figure 3:**
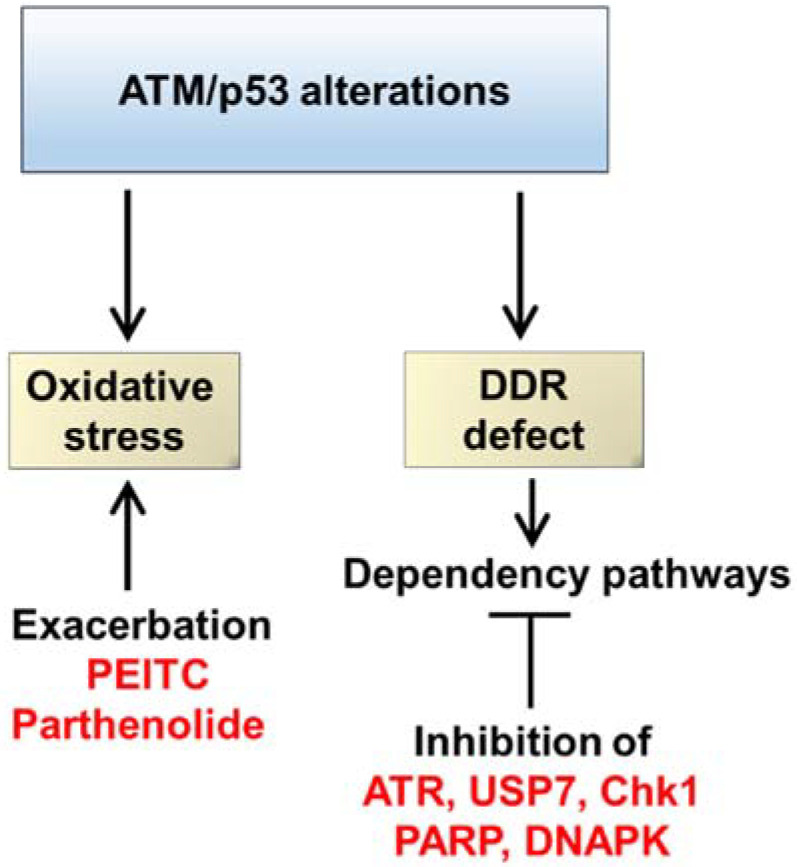
Exploiting cellular vulnerabilities induced by a defective p53 pathway. Alterations in the p53 pathway abrogate response to cellular stress. As a consequence, p53- or ATM-defective cells are sensitive to oxidative-stress-inducing agents such as PEITC or parthenolide. Abrogation of DNA damage response (DDR) leads to a number of cellular dependencies (addictions) in which tumor cells rely upon alternative pathways to repair DNA damage. Consequently, inhibition of these dependency pathways (i.e., ATR, USP7, PARP, or DNA-PK) leads to selective killing of p53 pathway-defective cells.

CLL cells with p53 pathway defects can also display functional addiction to other DNA repair proteins. Poly (ADP-ribose) polymerase (PARP) instigates the cellular repair of DNA single-strand breaks (SSBs) through a process termed PARylation, in which polymers of PAR are attached to itself and other proteins to generate a repair complex at the site of DNA damage [140,141]. Reminiscent of the effect of ATR inhibition, PARP inhibition leads to the enforced conversion of unrepaired SSBs into DSBs, which necessitates homologous recombination repair through ATM [71]. In a previous study, we demonstrated that the PARP inhibitor olaparib induced selective killing of *ATM*-defective CLL cells that accumulate catastrophic levels of DNA damage (Figure 3) [71]. This was corroborated by studies on *Eμ:TCL1*-driven autochthonous murine models of *ATM*-deleted CLL, showing exquisite and genotype-specific sensitivity to olaparib [142]. Our subsequent early-phase trial of olaparib in relapsed lymphoid cancers, including nine patients with CLL, confirmed acceptable tolerability of olaparib, with myelosuppression again being the most common toxicity. While on twice-daily olaparib, patients with ATM-pathway alterations displayed a longer median PFS of 83 days compared to 38 days among those with an intact ATM pathway [92].

Notwithstanding these findings, several studies have suggested that the utility of PARP inhibitors is not necessarily confined to CLL with *ATM* defects [143,144]. In a recent study, we demonstrated that the disruption of genes encoding ribonuclease H2, including *RNASEH2B,* which is frequently codeleted alongside miR-15a/16-1 in CLL cells harboring del(13q14) [145], confers sensitivity to PARP inhibition [144]. Such sensitization to PARP inhibitors is underscored by impaired ribonucleotide excision repair, with resultant abundance of embedded ribonucleotides in cells harboring ribonuclease H2 deficiency [144,146]. This leads to the accumulation of PARP-trapping lesions, which impede DNA replication, and are a prerequisite for PARP-inhibitor-induced cytotoxicity [144,147]. In CLL, we demonstrated that the degree of sensitivity to the PARP inhibitor talazoparib correlated with the number of *RNASEH2B* alleles lost [144]. More extensive studies are underway at our center to confirm the value of the *RNASHE2B* defect as an additional predictive biomarker of sensitivity to PARP inhibition beyond *ATM* loss.

Another compelling therapeutic target for CLL with *ATM* defect is DNA-PK (Figure 3), in light of its role in mediating NHEJ [148], the DSB-repair backup pathway of low fidelity that serves as an alternative to ATM-dependent homologous recombination [133]. A corollary of DNA-PK inhibition in ATM-deficient CLL cells is the accumulation of unrepaired DSBs due to the combined impairment of homologous recombination and NHEJ [72]. Subsequent resection and conversion of these DSBs into replication protein A (RPA)-coated single-stranded DNA repair intermediates trigger an activation of the ATR/Chk1 pathway, with resultant p53-dependent apoptotic response [72]. Preclinical studies have shown that the genetic and pharmacological abrogation of DNA-PK signaling, the latter using the DNA-PK inhibitors KU-0060648 and NU7441, leads to the selective killing of *ATM*-defective CLL cells [72,73]. Subsequently, the efficacy of CC-115, a dual TORK/DNA-PK inhibitor that simultaneously targets NHEJ and BCR signaling, was evaluated in individuals with *ATM*-defective relapsed/refractory CLL or small lymphocytic lymphoma (SLL), eliciting a partial response in 3 of 8 patients [93,149].

Ubiquitin specific protease (USP7), a deubiquitylase that is widely known for its role in negatively regulating p53 function by preventing the autoubiquitination and proteasomal degradation of MDM2 [150,151], also represents a legitimate therapeutic target in CLL. In p53-proficient tumor cells, USP7 inhibition induces p53-dependent apoptosis through reversing MDM2-mediated downregulation of p53, reminiscent of the effect of MDM2 inhibitors [151,152]. However, CLL cells with *TP53* or *ATM* defects are equally sensitive to USP7 inhibition (Figure 3) [74]. Accounting for this observation, we reported a p53-independent mechanism that operates in CLL cells whereby USP7 inhibition compromises homologous recombination, resulting in the accumulation of DNA damage that leads to DNA fragmentation and necrotic cell death via unrestrained PARylation [74]. Although unproven, we speculated that this could arise from the addiction of CLL cells to homologous recombination secondary to CLL-specific abnormalities within backup DNA repair pathways. Studies have also attributed the effect of USP7 inhibition to the role of USP7 in the p53-independent inactivation of PTEN, a tumor suppressor [153]. The evident preclinical efficacy of USP7 inhibitors such as HBX19818 in CLL thus provides rationale for future clinical evaluation of this therapeutic strategy.

Finally, there is substantial argument for the combined use of one or more DNA repair inhibitors. For instance, studies have reported the existence of a DNA-PK-Chk1 backup pathway that can mediate resistance to ATR inhibitors in tumor cells with lower levels of replication stress [154], arguing for the simultaneous targeting of ATR with DNA-PK or Chk1. The combined use of PARP and ATR inhibitors has also demonstrated synergism [155], which could potentially reduce the risk of developing resistance to either inhibitors; for example, through reversion mutations that restore or bypass homologous recombination or ATR signaling. However, the benefit of enhanced tumor cytotoxicity must be carefully balanced by the potential impact on systemic toxicity from such a combination approach. 

## 6. Harnessing the Immunogenicity of p53 Pathway Defects

CLL is a malignancy with a clinical course shaped as much by microenvironmental interactions as by genomic alterations. In spite of the acknowledged adverse prognostic impact of *TP53* mutations in CLL, not every patient with del(17p)/*TP53*-mutant MBL or early-stage CLL progresses to advanced disease [33,156]. In fact, among our recently reported cohort of 20 spontaneously regressing CLL cases, three harbored *TP53* mutations [1]. This serves to illustrate the cooperativity of genetic and microenvironmental factors in shaping the clinical course of patients with CLL. CLL cells suppress and co-opt the immune system to support their survival and proliferation [157,158,159]. Accordingly, the critical role of antitumor immunity and tumor-immune coevolution is also increasingly being recognized [5,160,161,162].

**Figure 4:**
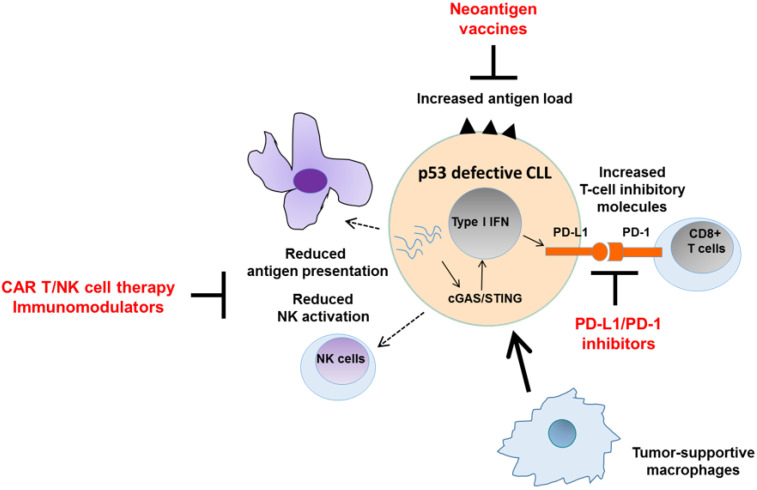
Targeting immune defects associated with a defective p53 pathway. p53 plays an important role in the regulation of antitumor immunity. p53 defective tumors evade immune response through multiple mechanisms, including impaired antigen presentation and T/NK cell activation, as well as macrophage reprogramming towards tumor-supporting phenotypes. In addition, the accumulation of unrepaired DNA damage with subsequent activation of cGAS/STING and type I interferon response could lead to upregulation of the T-cell inhibitory molecule PD-L1. These aspects of immune evasion can be counteracted by the use of various immunomodulatory agents. Defects in the p53 pathway are also associated with enhanced generation of neoantigens. This provides an opportunity for the generation of neoantigen vaccines as a strategy to eradicate p53-mutant tumor cells.

Mutated genes along the p53 pathway, including *TP53* and *ATM*, are known to have immunogenic potential and can elicit antitumor response as neoantigens [163,164,165]. Reports have also linked deleterious mutations that impair homologous recombination repair with greater neoantigen load [166,167]. On the other hand, the loss of wild-type p53 function contributes to immune suppression. First, wild-type p53 promotes antigen presentation on tumor cells by transcriptionally upregulating genes involved in major histocompatibility complex (MHC) class I peptide processing and presentation. These include *TAP1*, which mediates the transport of antigenic peptides from cytosol to endoplasmic reticulum for incorporation into assembled MHC class I molecules [168], and *ERAP1*, which trims antigenic precursors before MHC assembly [169]. Second, wild-type p53 promotes the tumor upregulation of natural killer (NK) cell-activating NKG2D ligands ULBP1 and ULBP2 [170], and induces miR-34a, which represses tumor expression of the T-cell inhibitory molecule PD-L1 [171]. Loss of wild-type p53 therefore contributes to impaired immune recognition of cancer cells and facilitates immune escape. Moreover, mutant p53 reprograms macrophages within the tumor microenvironment into a tumor-supportive phenotype [172]. Mutant p53 additionally disrupts cGAS/STING signaling [173], an important innate immune mechanism that detects tumor cytosolic DNA as signals of genomic instability, and transduces these signals into immune activity through type I interferon production [174,175,176]. Recent reports have also described the existence of a noncanonical ATM/p53-dependent, DNA-damage-responsive STING pathway, which is lost in tumors with ATM/p53 defects (Figure 4) [177].

These interactions between p53 pathway defects and tumor immunity represent a new frontier that can potentially be harnessed for CLL therapy. Immunotherapies such as chimeric antigen receptor (CAR) T/NK cell therapy and neoantigen vaccines in CLL have generated immense interest [94,95,96,97], but clinical efficacy has thus far been variable [178,179]. Effective ways to modulate the immunosuppressive CLL microenvironment to enhance immunotherapeutic efficacy are crucially needed. In this regard, there is considerable value in exploring p53 pathway targets as strategic avenues to enhance tumor immunity in CLL, possibly in combination with existing immunomodulatory agents such as PD-1/PD-L1 inhibitors, lenalidomide and ibrutinib (Figure 4) [157,179,180].

MDM2 inhibitors, for instance, have been shown to enhance tumor immunity and synergize with the PD-1/PD-L1 immune-checkpoint blockade [181,182]. The mechanistic basis of MDM2 inhibition on CD8^+^ T-cell function has recently been elucidated, and relates to the ability of MDM2 inhibitors to modulate stability of STAT5, which is essential for T-cell immunity [183]. Likewise, ATR inhibitors have been demonstrated to potentiate tumor immunity. This effect may be attributable in part to the ability of these inhibitors to attenuate CD8^+^ T-cell exhaustion through blocking tumor PD-L1 upregulation [184,185], and in part to the activation of a cGAS/STING-independent, RIG-I-dependent alternative innate immune pathway through concomitant loss of the G1/S and G2/M cell-cycle checkpoints in p53-deficient tumor cells upon ATR inhibition [186]. Finally, in tumors with homologous recombination repair defects, PARP inhibitors are also capable of augmenting PD-1/PD-L1 inhibitors through enhancing tumor immunogenicity and cGAS/STING signaling activity [187,188].

## 7. Conclusions and Future Perspectives

In this review, we provided an account of current and emergent strategies for p53 pathway targeting in cancer, highlighting their applicability and potential limitations for the treatment of CLL. From its modest beginnings, the field of p53 targeting has blossomed in recent years, with a wide range of therapeutic strategies currently being investigated. However, the development of many of these therapeutic strategies have thus far languished at the preclinical stage. As promising as many of them may appear from preclinical studies, few have successfully progressed beyond early-phase clinical trials, particularly in CLL. An arduous road thus lies ahead to translate laboratory findings into clinical benefit. The important questions to be answered relate to the place of these therapies among other CLL treatments and how they can be optimized.

In considering the value of p53 pathway targeting in the light of contemporary CLL treatments, one should focus especially on the considerable intertumoral and intratumoral heterogeneity that characterizes CLL [4,25,26,43]. Notwithstanding the transformative nature of therapies such as BCR signaling and Bcl-2 inhibitors, CLL remains incurable, with eventual disease relapse occurring due to the outgrowth of resistant subclones. Emergence of resistant mechanisms is dependent upon subclonal diversification and clonal evolution secondary to genomic instability, with p53 pathway defects being the major driver [26,46,189,190]. The value of therapeutic strategies targeting p53-specific dependencies lies in their ability to selectively eradicate the most genetically unstable CLL subclones that are frequently the source of disease persistence and the harbinger for disease relapse.

The major obstacles hindering the clinical development of p53-targeting therapies are their lack of single-agent clinical efficacy and relatively high level of toxicity, the latter being problematic particularly for CLL, with its demographic predilection for the elderly. The suboptimal clinical activity, often in spite of sound mechanistic basis and promising preclinical efficacy, can potentially be understood from the network of backup pathways and escape mechanisms that tumors can utilize in the face of p53 functional loss. The future for p53 targeting, in our view, thus lies in the combined use of synergistic therapies, both to improve therapeutic efficacy by reducing the likelihood of tumor evasion, and to reduce toxicity by potentially allowing lower drug doses to be used. Several recent studies have focused on the investigation of potential combinations involving p53/DDR-targeting treatments and BCR signaling or Bcl-2 inhibitors in CLL [60,190,191,192]. For instance, synergism between olaparib and ibrutinib was observed in del(11q) CLL underpinned by ibrutinib-mediated dysregulation of the homologous recombination repair protein Rad51 [193]. The notion of concomitant p53 activation and Bcl-2 inhibition is also exciting, and MDM2 inhibitors have been shown to promote Mcl-1 degradation in acute myeloid leukemia [194]. While these studies are invaluable, future work should also explore the combined use of different p53-targeting therapies, as well as the combination of p53-targeting therapies and immunotherapy.

We believe the accurate stratification of patients according to genetic biomarkers to be of particular importance in the clinical study of synthetically lethal and other p53-targeting therapies, in order to maximize therapeutic windows and minimize systemic toxicities. This also enables reduction in the potential duration of treatment and associated sublethal genotoxic exposures. Next-generation sequencing approaches can therefore be immensely useful in allowing the stratification of patients not only according to the presence or absence of a specific genetic mutation, but also the proportion of CLL cells harboring it, as well as the constellation of other genomic aberrations that may be present. It also permits the identification of additional predictive biomarkers of response and resistance that might help us further refine therapeutic stratification, thereby improving overall treatment outcome. In conclusion, the past two decades of research has certainly provided us with much of the scientific understanding underpinning therapeutic targeting of the p53 pathway, but also leaves us with a lot more to accomplish. The onus for us now is to build on this work, taking these treatments from the bench to the bedside.

## Figures and Tables

**Table 1:** Frequency of different p53 pathway alterations in patients with chronic lymphocytic leukemia.

Gene	Mutation Frequency	Deletion Frequency	Number of Patients Analyzed	Reference
	ND	18%	325	Döhner et al., 2000 [17]
	32%	4%	50	Stankovic et al., 2002 [13]
	12%	3%	155	Austen et al., 2005 [14]
*ATM*	ND	22%	330	Malcikova et al., 2009 [15]
	14.7%	30%	224	Skowronska et al., 2012 [19]
	8%	15%	160	Landau et al., 2013 [25]
	15%	22%	538	Landau et al., 2015 [26]
	ND	7%	325	Döhner et al., 2000 [17]
	12%	6%	50	Stankovic et al., 2002 [13]
	4%	ND	155	Austen et al., 2005 [14]
	5%	11%	400	Malcikova et al., 2009 [15]
*TP53*	8.5%	5%	328	Zenz et al., 2010 [16]
	7.6%	6%	529	Gonzalez et al., 2011 [27]
	15%	ND	309	Rossi et al., 2014 [28]
	11.5%	7%	635	Stilgenbauer et al., 2014 [29]
	13%	13%	160	Landau et al., 2013 [25]
	7%	6.3%	538	Landau et al., 2015 [26]

Abbreviations: ND, not determined.

**Table 2:** Pre-clinical studies with new therapeutic agents targeting defective p53 pathway.

Compound	Therapeutic Strategy	Cellular Effect	Reference
Targeting MDM2/p53 Axis			
Nutlin (RG7388)	MDM2 inhibition	Stabilization of wild-type p53, induction of p53 target genes and p53-mediated apoptosis in ATM deficient tumors	Kojima et al., 2006 [53] Coll-Mulet et al., 2006 [54] Saddler et al., 2008 [55] Ciardullo et al., 2019 [56]
Restoration of p53 tumor suppressor function			
Roscovitine (CYC202) Flavopiridol SNS-032 Dinaciclib	Pan-CDK inhibition	Suppression of p53-dependent pro-survival transcription in ATM- and p53-deficient tumors	Alvi et al., 2005 [57] Chen et al., 2005 [58] Chen et al., 2009 [59] Chen et al., 2016 [60]
PRIMA 1 APR-246	Refolding of mutant p53	Restoration of p53 wild- type properties and induction of cytotoxicity	Nahi et al., 2004 [61] Jaskova et al., 2020 [62]
Geldanamycin	HSP90 inhibition	Destabilization and degradation of mutant p53 and induction of cytotoxicity	Alexandrova et al., 2015 [63] Lin et al., 2008 [64]
Synthetic lethality			
PEITC Parthenolide	Depletion of cellular glutathione Induction of ROS	Exacerbation of oxidative stress to intolerable levels in p53- and ATM-deficient tumors	Liu et al., 2016 [65] Trachootham et al., 2008 [66] Agathanggelou et al., 2015 [67]
ATR inhibitor AZD6738 (ceralasertib)	Exploiting synthetically lethal interaction between ATR and ATM or p53	Exacerbation of replication stress in ATM- and p53-deficient tumors and induction of cellular death	Kwok et al., 2016 [68]
Chk1 inhibitor MU380	Exploiting synthetically lethal interaction between Chk1 and p53	Significant chemosensitization of *TP53*-mutant CLL cells and potentiation of nucleoside analog activity	Boudny et al., 2019 [69] Zemanova et al., 2016 [70]
PARP inhibitor olaparib	Exploiting synthetically lethal interaction between PARP1 and ATM	Exacerbation of unrepaired DNA damage and induction of cellular death	Weston et al., 2010 [71]
DNAPK inhibitors KU-0060648 and NU7441	Exploiting synthetically lethal interaction between DNAPK and ATM	Exacerbation of unrepaired DNA damage and selective killing of ATM-defective CLL cells	Riabinska et al., 2013 [72] Willmore et al., 2008 [73]
USP7 inhibitor HBX19818	Inhibition of HRR in ATM- and p53-deficient cells	Accumulation of DNA damage that leads to DNA fragmentation and necrotic cell death via unrestrained PARylation	Agathanggelou et al., 2017 [74]

Abbreviations: ROS, reactive oxidative species; HRR, homologous recombination repair.

**Table 3:** Clinical utility of new therapeutic strategies to target defective p53 pathway in CLL.

Compound	Clinical Trial	Observation	Reference
Nutlin RG7112	Phase I	Of 20 patients enrolled on the trial, one achieved partial response, whereas the majority maintained stable disease	Andreeff et al., 2016 [82]
Flavopiridol	Phase I	Successful induction of partial remission was observed in 45% of patients, with tumor lysis syndrome being the main dose-limiting toxicity	Byrd et al., 2007 [87]
SNS-032	Phase I	A single CLL patient responded out of 19 patients enrolled in the trial	Tong et al., 2010 [88]
Dinaciclib	Phase I/II Phase III	Partial response was observed in 28 of 52 patients with relapsed CLL in the first study [89], and in 8 of 20 patients in the second study [90]	Flynn et al., 2015 [89] Ghia et al., 2017 [90]
APR-246 (PRIMA-1)	Phase I	This study involved refractory AML and CLL patients. Clinical response was observed in a single CLL patient. APR-246 was well tolerated, with the most common adverse effects being of neurological nature	Deneberg et al., 2016 [91]
APR-246 + venetoclax	Phase I	Ongoing clinical trial	NCT04419389
ATR inhibitor ceralasertib + ibrutinib	Phase I	Ongoing clinical trial	NCT03328273
PARP inhibitor olaparib	Phase I	Nine CLL patients were enrolled in this trial. While on twice-daily olaparib, patients with ATM pathway alterations displayed a longer median PFS of 83 days compared to 38 days among those with an intact ATM pathway	Pratt et al., 2017 [92]
CC-115, a dual TORK/DNA-PK inhibitor	Phase I	Among 8 patients with ATM defective relapsed/refractory CLL or small lymphocytic lymphoma (SLL), a partial response was observed in 3 patients	Munster et al., 2019 [93]
CAR T/NK therapy	Pilot Phase I/II	Substantial elimination of CLL tumor cells	Porter et al., 2011 [94] Porter et al., 2015 [95] Liu et al., 2020 [96]
Neoantigen vaccines	Phase I	CD8+ T cells from vaccinated patients react against autologous CLL tumor	Burkhardt et al., 2013 [97]
