# Correction: Kwok et al. Targeting the p53 Pathway in CLL: State of the Art and Future Perspectives. *Cancers* 2021, *13*, 4681

**DOI:** 10.3390/cancers14020321

**Published:** 2022-01-10

**Authors:** Marwan Kwok, Angelo Agathanggelou, Nicholas Davies, Tatjana Stankovic

**Affiliations:** 1 Institute of Cancer and Genomic Sciences, University of Birmingham, Birmingham B15 2SY, UK; a.agathanggelou@bham.ac.uk (A.A.); n.j.davies@bham.ac.uk (N.D.); 2 Centre for Clinical Haematology, Queen Elizabeth Hospital Birmingham, Birmingham B15 2SY, UK

The authors wish to make the following corrections to their paper [1]:

1. There was an error in the original publication. Page 3. “Monoallelic *TP53* aberrations exert a more detrimental prognostic impact, compared to biallelic alterations.” This is incorrect. It should read: “Monoallelic *TP53* alterations exert a less detrimental prognostic impact, compared to biallelic alterations.”
2. There was an error in the original publication. Page 13. “In fact, among our recently reported cohort of 20 spontaneously regressing CLL cases, three harbored *TP53* mutations [2].” Reference [2] was erroneously cited. The correct reference here should be reference [1]. This sentence should read: “In fact, among our recently reported cohort of 20 spontaneously regressing CLL cases, three harbored *TP53* mutations [1].”
3. There was an error in the original publication. Page 14. “in combination with existing immunomodulatory agents such as the PD-1/PD-L1 inhibitors lenalidomide and ibrutinib” This sentence should read in “in combination with existing immunomodulatory agents such as PD-1/PD-L1 inhibitors, lenalidomide and ibrutinib.”
4. In the original publication, there was a mistake in Table 1: “Döhner” was misspelt as “Doner”. The corrected Table 1 appears below.

The authors apologize for any inconvenience caused and state that the scientific conclusions are unaffected. The original publication has also been updated.

## Figures and Tables

**Table 1:** Frequency of different p53 pathway alterations in patients with chronic lymphocytic leukemia.

Gene	Mutation Frequency	Deletion Frequency	Number of Patients Analyzed	Reference
*ATM*	ND	18%	325	Döhner et al., 2000 [17]
	32%	4%	50	Stankovic et al., 2002 [13]
	12%	3%	155	Austen et al., 2005 [14]
	ND	22%	330	Malcikova et al., 2009 [15]
	14.7%	30%	224	Skowronska et al., 2012 [19]
	8%	15%	160	Landau et al., 2013 [25]
	15%	22%	538	Landau et al., 2015 [26]
*TP53*	ND	7%	325	Döhner et al., 2000 [17]
	12%	6%	50	Stankovic et al., 2002 [13]
	4%	ND	155	Austen et al., 2005 [14]
	5%	11%	400	Malcikova et al., 2009 [15]
	8.5%	5%	328	Zenz et al., 2010 [16]
	7.6%	6%	529	Gonzalez et al., 2011 [27]
	15%	ND	309	Rossi et al., 2014 [28]
	11.5%	7%	635	Stilgenbauer et al., 2014 [29]
	13%	13%	160	Landau et al., 2013 [25]
	7%	6.3%	538	Landau et al., 2015 [26]

Abbreviations: ND, not determined.
